# A new way for punicalagin to alleviate insulin resistance: regulating gut microbiota and autophagy

**DOI:** 10.29219/fnr.v65.5689

**Published:** 2021-07-01

**Authors:** Yuan Cao, Guofeng Ren, Yahui Zhang, Hong Qin, Xin An, Yi Long, Jihua Chen, Lina Yang

**Affiliations:** 1 Department of Nutrition and Food Hygiene, Xiangya School of Public Health, Central South University, Changsha, China; 2 Children’s Medical Center, People’s Hospital, Hunan Province, Changsha, China

**Keywords:** punicalagin, insulin resistance, liver, gut microbiota, IKKβ/NF-κB, autophagy

## Abstract

**Background:**

Insulin resistance, defined as a diminished ability to respond to the stimulation of insulin, is the main line for a variety of metabolic-related diseases. Punicalagin (PU), a hydrolyzable tannin of pomegranate juice, exhibits multiple biological properties, including anti-oxidant, anti-cancer and anti-inflammatory activities.

**Objective:**

This research study aimed at determining the protective effect of PU on insulin resistance and to uncover the underlying mechanism based on the gut microbiota, IKKβ/NF-κB pathway, and autophagy.

**Design:**

An insulin resistance animal model was established using C57BL/6 mice fed with a high-fat diet (HFD) for 8 weeks. The model included two groups continuing a HFD for 12 weeks with or without administering via gavage with PU 20 mg/kg/day. Changes in fasting plasma glucose levels, fasting serum insulin levels, glucose and insulin tolerance, glycolipid metabolism, gut microbiota composition (16S rRNA gene sequencing), inflammatory responses, and autophagy in the liver were evaluated. Body weight gain, glycolipid metabolic disorder, liver injury, as well as systemic and hepatic insulin sensitivity, were significantly attenuated after supplementing with PU.

**Results:**

This research study revealed that PU alleviated HFD-induced glucose and lipid disorders, liver injury and insulin resistance; decreased the *Firmicutes/Bacteroides* ratio, decreased the abundance of *Coprococcus* and *Anaerotruncus,* and increased *Rikenellaceae*; and decreased serum and liver tumor necrosis factor-alpha and interleukin-1β levels, inhibited liver IKKβ and NF-κB phosphorylation; and increased liver autophagy-related proteins LC3-II, P62, and Beclin1, and increased the number of liver autophagosomes.

**Conclusion:**

PU can improve HFD-induced insulin resistance, improved liver glucose and lipid metabolism disorder and liver injury, and the potential mechanism is that PU inhibited the IKKβ/NF-κB inflammatory pathway by regulating gut microbiota homeostasis and up-regulating liver autophagy activity.

## Popular scientific summary

- Punicalagin alleviated high-fat diet induced glucose and lipid disorders, liver injury and insulin resistance.
- Punicalagin inhibited IKKβ/NF-κB inflammatory pathway by regulating gut microbiota homeostasis and up-regulating liver autophagy activity.

Insulin resistance is the main thread and common pathophysiological basis, which runs through various metabolic-related diseases, including hypertension and diabetes, which is a pathological state of decrease in insulin-mediated glucose uptake and utilization into the muscles and liver, leading to compensatory hyperinsulinemia (1). The liver, which is the main site of insulin resistance and a vital organ regulating body metabolism and insulin sensitivity (2), plays a key role in regulating metabolic homeostasis (3, 4).

IKKβ/NF-κB inflammatory pathway is the core mechanism linking inflammatory response and hepatic insulin resistance, which is particularly pivotal for regulating liver glucose and lipid metabolism (5). Nuclear factor-kappaB (NF-κβ) induces the release of amounts of transcription genes of proinflammatory cytokines (6), which alters insulin-mediated insulin receptor substrate (IRS) tyrosine phosphorylation, further leading to impaired binding of IRS to downstream PI3K and interfering with the insulin signaling cascades (7–9).

The gut microbiota may be a potential source of proinflammatory elements, which plays a decisive role in regulating energy homeostasis, chronic inflammation, and metabolic-related disorders like insulin resistance, and the interaction will affect the gut microbiota composition, which directly affects the intestinal immune homeostasis (10). As early as the 1990s, it was reported that inflammatory response is an important factor in the occurrence and development of insulin resistance. The disturbance of gut microbiota will activate the inflammatory response, which is attributed to the gut microbiota-derived lipopolysaccharide (LPS). A large amount of LPS are directly transported to the liver through the portal vein circulation, resulting in increased systemic LPS levels (11), which further initiates downstream inflammatory signal transduction, such as NF-κB signaling pathways. This then triggers a series of inflammatory reactions (12), which will accelerate the occurrence of insulin resistance.

Autophagy is a lysosomal degradation pathway, which maintains cell homeostasis by degrading or removing damaged proteins and organelles (13). An imbalance in high-fat diet (HFD)-induced gut microbiota leads to a state of excessive and/or persistent inflammation in our body, with tissues and organs being subjected to a high concentration of inflammatory mediators for a long time, which destroys the cell homeostasis. To maintain intracellular homeostasis, it is inevitable that autophagy participates in the suppression of inflammatory response (14). It was reported that autophagy could prevent excessive inflammatory reactions in autophagy-inhibiting mice, which produced more inflammatory cytokines than the normal mice and more susceptible to sepsis (15).

Pomegranates are a rich source of polyphenols that are the main phytochemicals present in pomegranate peels, which mainly include punicalagin (PU), ellagic acid and gallic acid. During the metabolism of gut microbiota, PU mainly generates urolithin A, the formation of which is closely connected to the structure of human and animal gut microbiota composition (16). Urolithin A can significantly inhibit interleukin-1β (IL-1β) or tumor necrosis factor-alpha (TNF-α)-induced inflammatory response in colon fibroblasts (17) to improve the anti-inflammatory activity, thereby improving the function of the intestine and the growth performance of animals. Several studies have shown that polyphenols, such as cranberry polyphenol extracts and resveratrol, can improve obesity-related disorders, which may be linked with gut microbiota homeostasis, inflammation and autophagy (18–20). In this study, we aimed to evaluate the anti-insulin resistance effects of PU on HFD-induced insulin resistance, and to declare whether gut microbiota homeostasis, IKKβ/NF-κB pathway and liver autophagy are related to the improving effects of PU on insulin resistance and the underlying mechanism.

## Materials and methods

### Animal experiments

Six- to eight-week-old male C57BL/6 mice (20 ± 2 g) were used to explore the protective effects of PU (purity > 98%; 65995-63-3, Chengdu Herbpurify Co. Ltd.Chengdu, China) and the possible mechanism on insulin resistance. After acclimatization for a week, mice were randomly divided into normal diet (D12450B, Research Diets, New Brunswick, NJ) group (CON group, 10 mice) and HFD (D12492, Research Diets, New Brunswick, NJ) group (HFD group, 20 mice). After an 8-week exposure to the respective diets, fasting plasma glucose, insulin, intra-peritoneal glucose tolerance test (IPGTT) and insulin tolerance test (ITT) assay were carried out. There was a significant difference in HOMA-IR index, AUC_GTT_, and AUC_ITT_ between the CON group and the HFD group, which was considered as the insulin resistance model was established. Then HFD group mice were randomly divided into two groups (*n* = 10): insulin resistance mice fed with water only (IR group) and insulin resistance mice administered with an oral gavage of 20 mg/kg/day PU, where PU was dissolved in distilled water (PU group). Twelve weeks later, mice were subjected to fasting plasma glucose, IPGTT and ITT assays before being sacrificed. Their serum samples were collected for biochemical analysis. Liver samples were harvested for Western blotting, biochemical tests, or histochemical examination. All experiments were carried out with the approval of the Institutional Animal Care and Use Committee at the Xiangya Medical College, Central South University. Homeostasis Model Assessment of Insulin Resistance (HOMA-IR) index = fasting insulin (mIU/L) × fasting glucose (mmol/L) / 22.5 (21).

### GTT and ITT

At the end of weeks 8 and 20, after 12-h fasting, mice were intraperitoneally injected with glucose (2.0 g/kg) for the intra-peritoneal glucose tolerance test (GTT); after 6-h fasting, mice were intraperitoneally injected with insulin (0.75 IU/kg) (Novolin R; Novo Nordisk, Bagsværd, Denmark) for the ITT. Blood glucose levels were detected before and at 30, 60, 90 and 120 min after the injection.

### Liver histology

Sections of 4% paraformaldehyde-fixed liver samples were taken for histological evaluation. Liver pathological was evaluated by hematoxylin-eosin (H&E) staining. Liver lipid accumulation was detected by Oil Red O (ORO) staining. Liver glycogen deposits were detected by Periodic acid-Schiff (PAS) staining. All the images were captured under a Motic microscope BA310 (Ted Pella Inc., Los Angeles, CA).

### Biochemical analysis

According to the corresponding manufacturer’s instructions, Enzyme Linked Immunosorbent Assay (ELISA) kits (ABclonal Biotechnology Co., Ltd, Wuhan, China) of mouse insulin, IL-6 and TNF-α were employed for serum. Reagent kits (Jiancheng Bioengineering Institute, Nanjing, China) of ALT and AST were employed for biochemical detection of serum, Reagent kits (Jiancheng Bioengineering Institute, Nanjing, China) of triglyceride (TG), free fatty acid (FFA) and glycogen content were employed for biochemical detection of liver tissue.

### Transmission electron microscopy

Sections of 2.5% glutaraldehyde liver samples were taken for autophagy evaluation. All the images were captured under a Zeiss EM 900 transmission electron microscope (TEM).

### Western Blotting

Liver tissue proteins were extracted by Radio Immunoprecipitation Assay (RIPA) lysis buffer containing protease inhibitors (Beyotime, Shanghai, China) and qualified by Bicinchoninic Acid Assay (BCA) protein assay reagents (Boster, Wuhan, China). sodium dodecyl sulfate polyacrylamide gel electrophoresis (SDS-PAGE) was used to separate samples, and the proteins were transferred to polyvinylidene fluoride (PVDF) membranes (Immobilon; Millipore, Bedford, MA, USA) using a wet electrophoretic transfer. Membranes were blocked room temperature (RT), 1 h) in 5% non-fat milk powder dissolved in Tris-buffered saline (20 mM Tris base, 0.5 M NaCl, pH 7.5) and 0.1% Tween-20 (TBST). Then the membrane was incubated with primary antibodies including β-actin (60008-1-Ig, Proteintech), IKK (A0714, ABclonal), P-IKK (AF3014, Affinity), P65 (A2547, ABclonal), P-P65 (A0475, ABclonal), TNF-α (A0277, ABclonal), IL-1β (A17361, ABclonal), P62 (55274-1-AP, Proteintech), LC3A/B (AF5402, Affinity), Beclin1 (11306-1-AP, Proteintech) (4°C, overnight), respectively. After TBST washed three times, the membrane was incubated with secondary antibody Horseradish Peroxidase (HRP)-conjugated anti-rabbit or anti-mouse IgGs (ZSGB-BIO Biotechnology Co. Ltd, Beijing, China) (RT, 1h). After washing three times with TBST, membranes were detected by BosterECL Star reagent (AR1170, BOSTER).

### Analysis of gut microbiota

Twenty-four hours after the last intragastric administration, the feces of each group were collected in 2 mL dry sterilized EP tubes and stored in a refrigerator at −80°C for testing. The fecal samples were sent to Shanghai Personal Biomedical Technology Co. Ltd. for 16S rRNA high-throughput sequencing based on Illumina MiSeq. Using forward primers: 308F(5′-ACTCCTACGGGAGGCAGCA-3′), 806R (5′-GGACTACHVGGGTWTCTAAT-3′) to amplify the V3–V4 region of bacterial 16S rRNA genes. QIIME (v1.8.0) and R packages (v3.5.1) was used to perform sequence processing. Differences in taxa abundance were assessed by one-way analysis of variance (Fisher’s post hoc test; Metastats). Linear discriminant analysis effect size (LEfSe) analysis was performed on the Galaxy online module.

### Statistical analysis

The data were expressed as means ± SD, and each condition was performed at least three times. Statistical analysis was carried out using unpaired Student’s *t*-test or one-way analysis of variance, and statistical significance was assessed by SPSS 18.0. *P* < 0.05 was considered to be statistically significant.

## Results

### High-fat diet-induced insulin resistance model

**Fig. 1:**
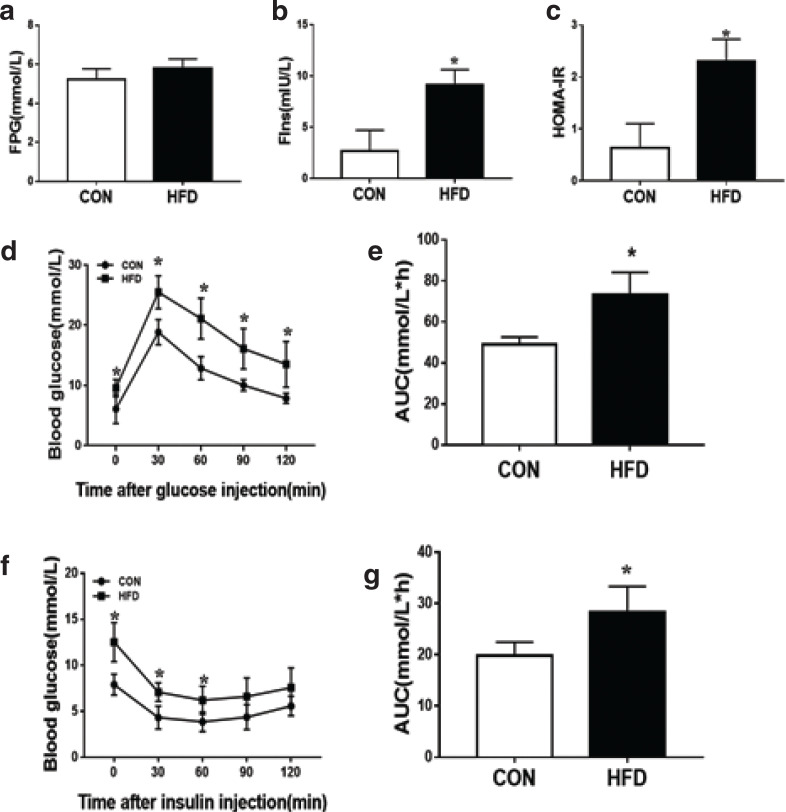
High-fat diet-induced insulin resistance model. FPG (a), Fins (b), HOMA-IR (c), IPGTT (d), AUC of IPGTT (e), ITT (f), AUC of ITT (g). Values indicate mean ± SD. (*n* = 6–7) *P < 0.05, compared with the CON group.

After 8 weeks of HFD, compared with the CON group, there was a significant increase in fasting serum insulin concentration in the HFD group, and HOMA-IR had consistent results; there was no significant difference in fasting blood glucose levels between the two groups (Fig. 1a–c). We also performed GTT and ITT, and the area under the curve (AUC) was calculated to quantify glucose and insulin tolerance. GTT analysis showed that after animals received glucose load through intraperitoneal injection, plasma glucose concentrations were significantly greater in the HFD group and peaked at 30 min (Fig. 1d), and were kept higher at all the time points compared with the CON group. AUCs (Fig. 1f) of glucose concentration during GTT showed that HFD group markedly increased compared with the CON group. ITT analysis showed that after animals were intraperitoneally injected with insulin load through intraperitoneal injection, plasma glucose concentrations were significantly greater in the HFD group and peaked at 60 min (Fig. 1d), and remained higher at all the time points compared with the CON group. AUCs (Fig. 1f) of glucose concentration during ITT showed that HFD group markedly increased compared to the CON group. These results suggested that the HFD-induced insulin resistance model was successfully established after an 8-week HFD. The HFD group was randomly divided into two groups, IR group and PU group, which was regarded as a model group and a treatment group (20 mg/kg/day administered an oral gavage of PU), respectively.

### Effect of PU on high-fat diet-induced insulin resistance

**Fig. 2:**
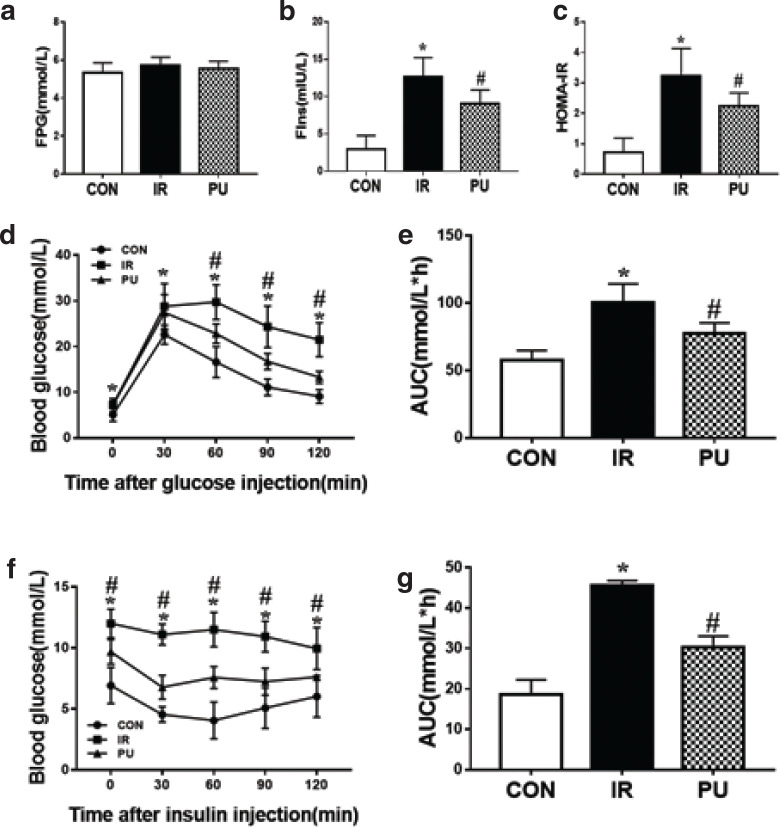
Effect of PU on high-fat diet induced insulin resistance. FPG (a), Fins (b), HOMA-IR (c), IPGTT (d), AUC of IPGTT (e), ITT (f), AUC of ITT (g). Values indicate mean ± SD. (*n* = 6–7) *P < 0.05, compared with the CON group, ^#^*P* < 0.05, compared with the IR group.

After 12 weeks of PU supplementation, there was a significant decrease in both fasting serum insulin levels and HOMA-IR compared with the IR group, while fasting plasma glucose levels showed no significant difference among the three groups (Fig. 2a–c). GTT and ITT analysis indicated that PU markedly improved HFD-induced insulin resistance, which was comparable with the IR group (Fig. 2d, f). Compared with the IR group, PU supplementation significantly strengthened the insulin-induced glucose-lowering effect. Meanwhile, AUCs of glucose concentration during GTT and ITT (Fig. 2e, g) both significantly decreased in PU groups, compared with those in the IR group, thus indicating improved glucose tolerances. In addition, the fasting glucose levels remained unchanged in the IR and PU groups compared with the CON group. Also, PU supplementation decreased serum insulin levels and HOMA-IR in IR mice (Fig. 2c). These results revealed that PU supplementation played a significant role in alleviating HFD-induced insulin resistance.

### Effects of PU on liver

**Fig. 3:**
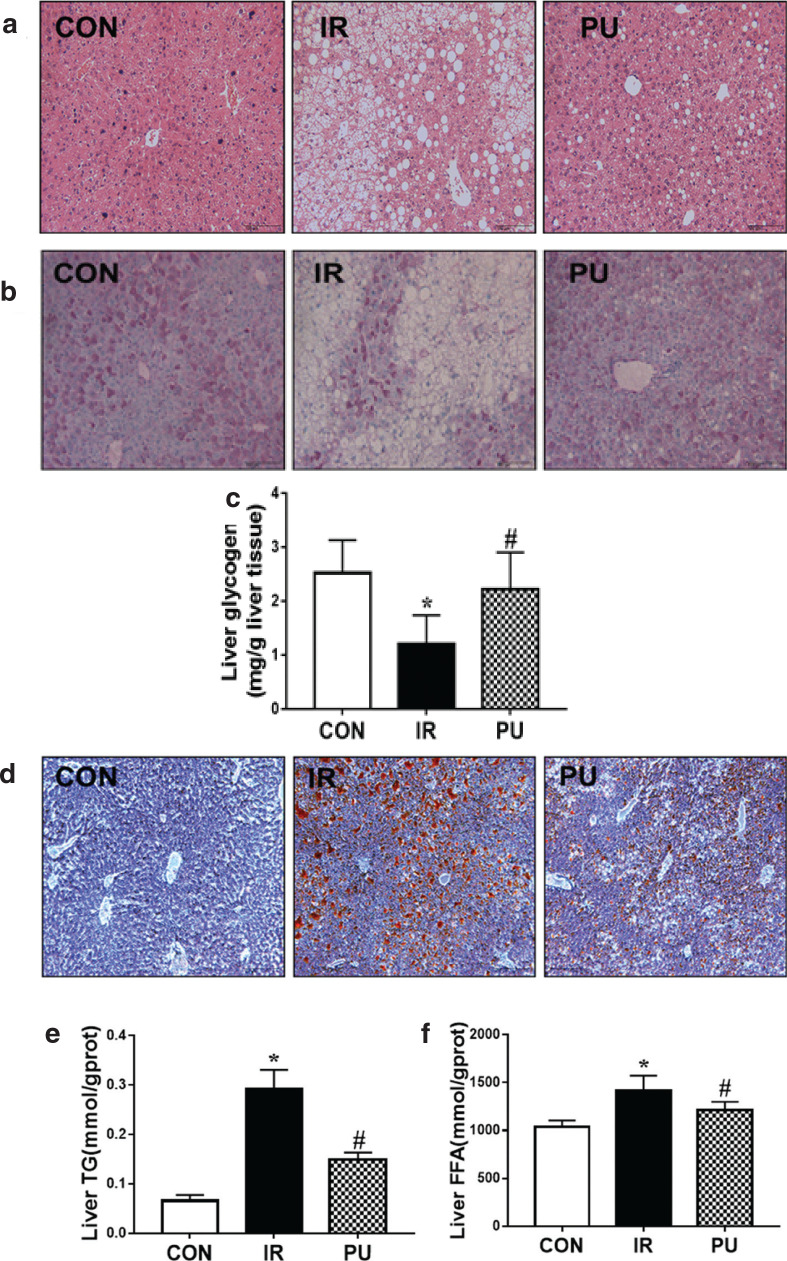
Effect of PU on the body weight, liver injury and glucose and lipid metabolism. Effects of PU upon hepatic pathologic change (a) determined by H&E staining, hepatic glycogen content (b) determined by PAS staining, hepatic lipid accumulation determined by ORO staining (d). Magnification: 100x. Liver tissue biochemical markers: Liver glycogen (c), Liver FFA (e), Liver TG (f). Values indicate mean ± SD. (*n* = 6–7) *P < 0.05, compared with the CON group, ^#^*P* < 0.05, compared with the IR group.

As shown in Table 1, PU significantly restored HFD-induced increase in body weight and liver weight ratio. HFD resulted in liver injury compared with the IR group, and PU-treated mice significantly decreased the serum Alanine aminotransferase (ALT) activity. There was no significant difference in serum Aspartate aminotransferase (AST) activity among all groups. H&E staining (Fig. 3a) revealed that the liver architecture of IR group mice changed compared with the CON group, including cellular degeneration, hepatocyte necrosis and cytoplasmic vacuolation, and PU supplementation reversed HFD-induced changes. These results revealed that PU effectively inhibited HFD-induced body gain and liver weight ratio gain and markedly inhibited HFD-induced liver injury.

**Table 1:** Body weight, liver weight ratio and serum ALT and AST (*x* ± s)

	CON	IR	PU
Body weight (g)	33.36±1.91	50.40±1.80*	42.75±3.19#
Liver weight ratio (%)	3.11±0.24	4.11±0.32*	2.85±0.29#
ALT (U/L)	107.03±5.02	165.19±10.16*	137.86±9.63#
AST (U/L)	81.08±6.85	85.67±18.20	82.98±11.18

* P < 0.05, compared with the CON group

# P < 0.05, compared with the IR group (n = 6–7)

In addition, PAS and ORO staining were carried out to analyse glycogen storage and lipid accumulation in liver, respectively (Fig. 3b, d). HFD led to an obvious decrease of glycogen in liver. PU supplementation markedly enhanced glycogen storage in liver compared with the IR group (Fig. 3c). A significant accumulation of lipids was observed in the liver of HFD mice via ORO staining. Besides, PU supplementation markedly reduced lipid accumulation (Fig. 3d). These results were in accordance with the biochemical results of liver tissue. There was a significant decrease in the liver glycogen content in the IR group compared with the CON group (Fig. 3b), and PU supplementation reversed this effect. The levels of FFA and TG in liver increased remarkably in the IR group (Fig. 3e, f), while PU supplementation decreased FFA and TG accumulation in liver. Therefore, histopathological and biochemical results suggested that PU alleviated insulin resistance and prevented liver from HFD-induced hepatic glucose and lipid metabolism disorders.

### Effect of PU on high-fat diet-induced IKKβ/NF-κB activation and inflammatory mediator release

**Fig. 4:**
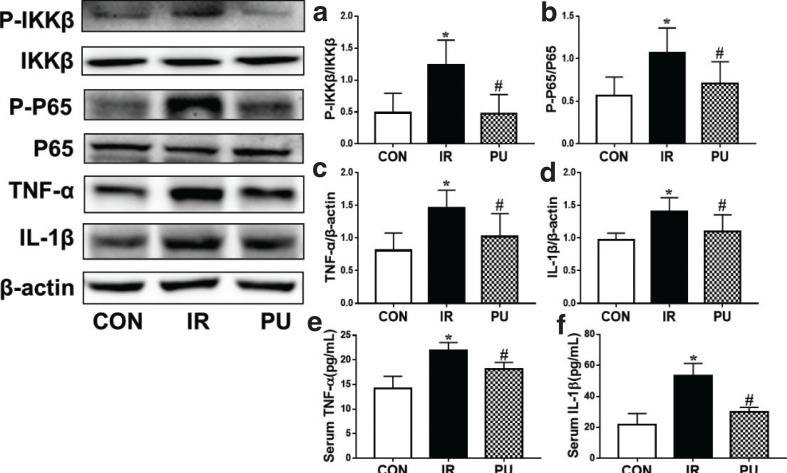
Effect of PU on high fat diet-induced IKK(β/NF-κB activation and inflammatory mediators release. Ratios of liver P-IKKβ/IKKβ (a), P-P65/P65 (b), TNF-α (c), IL-1β (d) determined by western blotting, Sérum TNF-α (e), IL-1β (f) release, determined by ELISA. Values indicate mean ± SD. (*n* = 6–7) *P < 0.05, compared with the CON group, ^#^*P* < 0.05, compared with the IR group.

Inflammation is closely associated with the development of insulin resistance, and IKKβ/NF-κB inflammatory pathway is the key point of junction between hepatic inflammation and insulin resistance. NF-κB, a nuclear transcriptional activator, can induce the release of amounts of transcription genes of proinflammatory cytokines, including TNF-α, interleukin-6 (IL-6) and IL-1β (5). To investigate the possible mechanism of how PU improves insulin resistance, IKKβ/NF-κB activation in the livers was measured by Western blotting. The phosphorylation levels of IKKβ and NF-κB P65 increased obviously in the IR group, and PU supplementation remarkably decreased the ratio of P-IKKβ/IKKβ and P-P65/P65 (Fig. 4a, b). Subsequently, liver and serum inflammatory mediators TNF-α and IL-1β release were detected by ELISA (Fig. 4c–f). The levels of TNF-α and IL-1β were markedly increased in the IR group, and it was reversed by the treatment of PU. Decreased levels of TNF-α and IL-1β were observed in liver and serum in the PU group.

### Effect of PU on gut microbiota

**Fig. 5:**
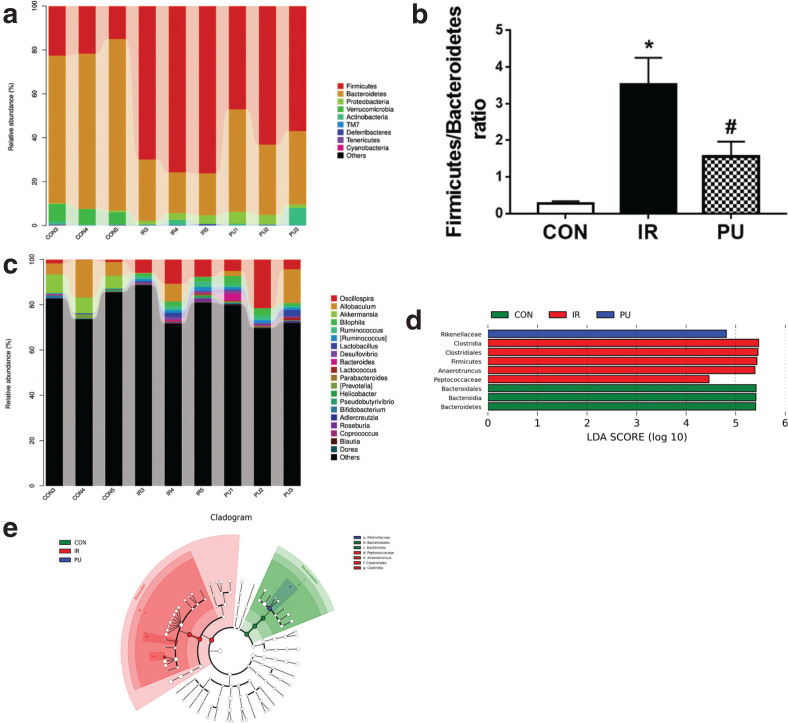
Effect of PU on gut microbiota. Relative abundance distribution at bacterial phylum levels (a). Comparison of firmicutes/bacteroidetes ratio in different groups (b). Top 20 enriched genera categories (c). LDA at the level of phylum, class, order and genera (d). Cladogram generated by LEfSe analysis (e). Values indicate mean ± SD. (*n* = 3) *P < 0.05, compared with the CON group, ^#^*P* < 0.05, compared with the IR group.

At the phylum level (Fig. 5a), the dominant species in each group include *Firmicutes* and *Bacteroidetes*. The IR group increased the abundance of *Firmicutes* and decreased the abundance of *Bacteroides* compared with the CON group. PU supplementation decreased the abundance of *Firmicutes* and increased *Bacteroides*. Owing to the importance of the *Firmicutes*-to-*Bacteroides* (*Firmicutes/Bacteroidetes*, F/B) ratio in insulin resistance, the F/B of each group was calculated (Fig. 5b). It was observed that the F/B ratio significantly increased in the IR group (*P* < 0.05), while the F/B ratio significantly decreased in the PU group (*P* < 0.05).

At the genus level (Fig. 5c), compared with the CON group, there was an increase in the abundance of *Desulfovibrio, Oscillospira, Bilophila, Ruminococcus, Lactococcus [Ruminococcus], Anaerotruncus, Adlercreutzia, Blautia, Coprococcus* and *Dorea*, and a decrease in the abundance of *Akkermansia* and *Allobaculum* in the IR group; PU increased the abundance of Coprococcus and Anaerotruncus.

LEfSe analysis is a classical dimensionality reduction method of linear discriminant analysis (LDA), which analyzes the differences between groups and finds out the different species (Fig. 5d). From the results of LDA, we have drawn the LEfSe multi-level species hierarchy tree (Fig. 5e). At different classification levels, the relative abundance of *Firmicutes* in the IR group was much higher than that of the other two groups, and the main microbiota includes *Clostridia* class, *Clostridia* order, *Peptococcaceae* and *Anaerotruncus* belonging to *Clostridium*. While *Bacteroides* phylum, Class, and Order were widely distributed in the CON group, *Rikenellaceae* belonging to the *Bacteroides* order was widely distributed in the PU group.

### Effect of PU on liver autophagy

**Fig. 6:**
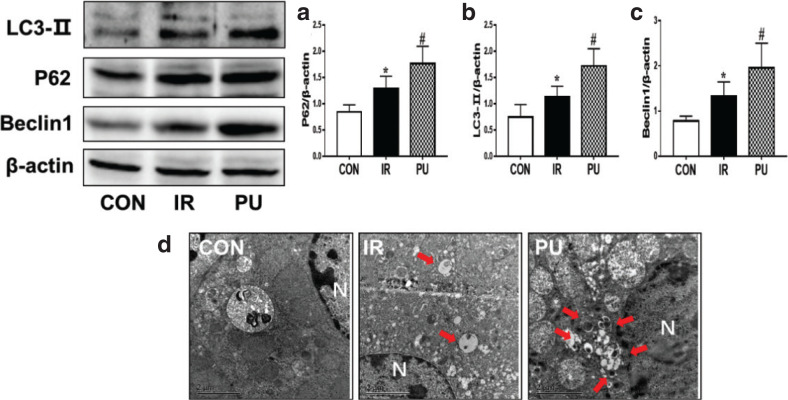
Effect of PU on liver autophagy. Ratios of liver p62 (a), LC3- II (b), Beclin1 (c), determined by western blotting. Autophagosomes analysis, determined by TEM (d) (The red points to the autophagosome, *N* indicates the nucleus, Scale Bar=2 μm), values indicate mean ± SD. (*n* = 6–7) *P < 0.05, compared with the CON group, ^#^*P* < 0.05, compared with the IR group.

To evaluate whether autophagy was associated with insulin resistance, autophagy markers (LC3-II, a marker for autophagy induction; P62, an indication of autophagic clearance; and Beclin1, a protein for autophagy initiation) were determined in the livers by Western blotting. Compared with the CON group, the expression of LC3, Beclin1, and P62 was significantly increased in the IR group. After supplementation with PU, the expression of LC3-II, P62 and Beclin1 (Fig. 6a-c) was remarkably elevated compared with the IR group. Meanwhile, TEM results (Fig. 6d) revealed that the numbers of autophagosomes in the CON group were less than that in the IR group. There was an increased autophagosome formation in the PU group compared with the IR group. These results suggested that HFD and PU supplementation both enhanced autophagy activation in livers.

## Discussion

PU, during the metabolism of gut microbiota, mainly produces urolithin A (16). Zhao et al. (22) showed that PU and urolithin A could alleviate LPS-induced colitis activation, and reduce intestinal injury and regulate gut microbiota disorder to inhibit systemic inflammation. Urolithin A can inhibit the NF-κB inflammatory pathway activation, thereby inhibiting the acute inflammation caused by IL-1β in human colon fibroblasts (17). This study found that PU alleviated HFD-induced glucose and lipid metabolic disorders, changed gut microbiota composition, inhibited the IKKβ/NF-κB signaling pathway and inflammatory cytokine secretion, and induced liver autophagy, which might be the crucial mechanism to attenuate HFD-induced insulin resistance.

Insulin resistance is a decreased ability of insulin-mediated glucose disposal into the muscles and liver, resulting in compensatory circulating hyperinsulinemia (23).HOMA-IR is widely used in the detection of insulin resistance in *vivo*, which is a simple and practicable method to replace the gold standard of insulin resistance-insulin-glucose clamp test (24). Here, we applied HFD in C57/BL6 mice to mimic an insulin resistance status *in vivo* to explore the anti-insulin resistance effect of PU and the underlying mechanism. In this study, PU can not only alleviate the inflammatory response but also induce hepatic autophagy to further exert its hepatoprotective effect, thereby improving hepatic glucose and lipid metabolic disorders.

In this study, we chose 20 mg/kg/day PU as the intervention dose. Peng et al. (25) showed that mice with LPS-induced acute lung injury intraperitoneally injected with 12.5 and 25 mg/kg/day PU significantly inhibited the activation of NF-κB pathway and the release of inflammatory factors. Gavage of 15 and 30 mg/kg/day PU for 7 days significantly inhibited the release of inflammatory factors during myocardial ischemia-reperfusion injury in Wistar rats (26). Combined with the results of our preliminary experiments, the HFD mice were given 15 mg/kg/day PU by intragastric administration, in which it was observed that the release of inflammatory factors was decreased and the IR symptoms were alleviated. We found that PU intake reduced HFD-induced weight gain, AST level, and the HE staining showed that PU ameliorated lobular inflammation and diffuse ballooning degeneration in hepatocytes, which demonstrated the potential effect of PU in preventing HFD-induced liver inflammation and injury. However, the ALT level and FPG of HFD-induced mice had no significant difference compared with normal diet mice, the results were consistent with previous studies (27, 28), and it was probably attributed to the body’s natural protective compensatory response under high-fat conditions. At the same time, this might indicate that HFD-induced insulin resistance was at the early stage and further liver function was not severely deteriorated. It is quite important that insulin regulates glucose uptake, utilization and storage to maintain blood glucose homeostasis (2). In this study, we found that PU improved HFD-induced insulin resistance, as supported by the decreased fasting serum insulin levels, HOMA-IR index, and the results of the GTT and ITT, indicating that insulin sensitivity had been improved.

Liver, the main site where insulin resistance occurs, is a vital organ that regulates metabolism and insulin sensitivity (29, 30), and plays a key role in controlling insulin sensitivity. The results of this study showed that PU can significantly reduce liver TG and FFA levels and reduce liver lipid accumulation, thus resulting in the improvement of liver lipid metabolism disorders. Increased hepatic TG and FFA content, and large lipid droplets were observed with ORO staining in the IR group, and PU intake reversed the lipid accumulation in hepatocytes. Similarly, the results from both biomechanical staining and PAS revealed that PU supplementation remarkably reversed the decrease of HFD-induced hepatic glycogen content. The results also suggested that PU intake remarkably improved HFD-induced glucose and lipid metabolism disorders, which was the manifestation of insulin resistance.

Inflammation is one of the main inducements of insulin resistance, and the disturbance of gut microbiota leads to increase in intestinal permeability, thus allowing large amounts of LPS to enter blood circulation and subsequently activating systemic inflammatory responses. The IKKβ/NF-κB pathway, one of the classical inflammatory pathways, releases a large number of inflammatory cytokines, thus leading to the failure of target organs, including liver, muscles, and pancreas, to respond normally to the action of insulin, resulting in insulin resistance (31). Studies reported that the absence of TNF-α improved insulin sensitivity in obese mice (32), and plasma IL-1 is an important inflammatory marker of systemic insulin resistance (33). In this study, we observed that PU supplementation remarkably decreased gut microbiota disturbance-induced release of TNF-α and IL-1β, thus inhibiting the IKKβ/NF-κB inflammatory pathway phosphorylation and reducing the production of TNF-α and IL-1β, thereby improving insulin resistance.

Gut microbiota has significant implications in the pathogenesis of obesity, inflammation and insulin resistance. Long-term HFDs can damage the intestinal mucosa and destroy the gut microbiota composition, ultimately resulting in immune response and metabolic disorders, and inducing a series of disorders like insulin resistance (34). Therefore, maintaining the gut microbiota composition and adjusting the distribution of flora are important ways of inhibiting inflammatory pathways and insulin resistance. The results of this study revealed that at the levels of bacterial phylum, PU supplementation increased the abundance of *Bacteroides* and decreased the *Firmicutes/Bacteroides* ratio, which played a significant role in the HFD-induced metabolic disorders (35). In addition, most bacteria belonging to the *Bacteroides* can produce polysaccharide A, thus regulating the immune response (36).

At the levels of bacterial genera, IR group increased the abundance of *Desulfovibrio, Oscillospira, Bilophila, Ruminococcus, Lactococcus, [Ruminococcus], Anaerotruncus, Adlercreutzia, Blautia, Coprococcus* and *Dorea*, and decreased the abundance of *Allobaculum* and *Akkermansia*; PU intake decreased the abundance of *Coprococcus* and *Anaerotruncus*. Velázque et al. (37) showed that HFD increased the abundance of *Dorea, Firmicutes, Adlercreutzia, Ruminococcus* and *Coprococcus*. Di Luccia et al. (38) observed that high-fructose diet increased the abundance of *Ruminococcus* and *Coprococcus*. After treating with antibiotics or fecal transplantation, the abundance of these two bacteria significantly reduced, thus inhibiting inflammation and insulin resistance, which indicates that *Ruminococcus* and *Coprococcus* are related to metabolic syndrome. Several strains of *Blautia* can promote the release of inflammatory factors, such as TNF-α. *Desulfovibrio* can produce a large amount of LPS, and the beneficial bacterium *Allobaculum* can produce butyrate, thus providing energy to the intestine cells to protect the intestinal barrier and reduce the LPS outflow (39). The evidence shows that gut microflora alteration in the IR group may be directly related to the increased inflammation. PU can significantly reduce the abundance of *Coprococcus* and conditioned pathogen *Anaerotruncus*, thus inhibiting inflammation and insulin resistance by regulating the gut microbiota composition.

LefSE analysis indicated that the abundance of *Firmicutes* in the IR group was much higher than that of CON and IR group, indicating that HFD-induced insulin resistance was closely related to the increased abundance of *Firmicutes*. And it was observed that *Clostridia* class, *Clostridia* order, *Peptococcaceae* and *Anaerotruncus* belonging to Clostridium played a major role in the *Firmicutes*. The *Clostridiales* order is closely related to host inflammatory reactions and comprises various kinds of bacteria, which are important pathogens related to production of botulinum toxins and fatal infections. The increase in the abundance of *Anaerotruncus* is directly associated with the increase of aging-induced inflammatory cytokines (40). In inflammatory diseases, such as chronic prostatitis and colitis, there was an increase in the abundance of *Peptococcaceae* (41, 42). The gut microbiota alteration in the IR group was consistent with the activation of inflammatory pathways and the release of inflammatory cytokines. The *Rikenellaceae* bacteria belonging to Bacteroides may have important implications for the anti-IR effect of PU, which increases the levels of butyrate to protect the function of mucosal barrier and delay the pathogenesis of metabolic diseases. Alard et al. (43) reported that HFD increased the abundance of *Rikenellaceae* bacteria in mice, accompanied by reduced insulin sensitivity. The results of this study demonstrated that PU increased the abundance of the *Rikenellaceae*, suggesting that PU may improve HFD-induced insulin resistance and glycolipid metabolism disorders by improving the makeup of gut microbiota.

Autophagy, a lysosomal-dependent degradation pathway, is characterized by the formation of autophagic vesicles, which has important implications for maintaining cellular homeostasis (44). Studies have shown that diet was able to change various cellular processes, especially autophagy, and autophagy may be beneficial in suppressing inflammation responses (45). It was reported that autophagy decreases pro-inflammatory signaling, such as IKKβ/NF-κB pathway, by eliminating damaged organelles, degrading pro-inflammatory signaling molecules, and controlling the production and release of inflammatory cytokines (46). Therefore, we speculated that autophagy was involved in the inhibiting effects of PU on inflammation, thereby improving insulin resistance.

Autophagy was associated with the key markers (47): an elevated expression of LC3-II and P62 and enhanced expression of Beclin1 in the IR and PU groups in livers. P62 is an autophagy adaptor for protein degradation, along with LC3-II, both of which are associated with autophagosome formation (48). Sometimes, the expression of P62 increased when autophagy is enhanced, and this might be because P62 production was higher than P62 degradation (49). Our study results worked in concert with Xu et al.’s (50) investigations. In addition, these results were in accordance with TEM results, which showed that the number of autophagosomes increased. Overall, these results revealed that autophagy was enhanced in the IR and PU groups. Therefore, we supposed that PU-upregulated autophagy activation might protect HFD-fed mice from insulin resistance. In this study, we reported that autophagy was characterized as a compensatory response to prevent HFD-induced lipid accumulation and insulin resistance. The mice were still in the early stage of insulin resistance, at that time, liver function was not severely damaged, and the compensatory protective mechanism was consistent with the changes in FPG and AST levels. To sum up, these results suggested that PU induced autophagy to improve insulin resistance, which was possibly by inhibiting the IKKβ/NF-κB inflammatory pathway.

In the study, PU improved HFD-induced insulin resistance and inhibited the activation of IKKβ/NF-κB inflammation pathway. The disturbance in HFD-induced intestinal flora resulted in the activation of inflammatory response, and the IKKβ/NF-κB inflammatory pathway is the core mechanism linking inflammatory response and hepatic insulin resistance. In addition, autophagy participated in the regulation of inflammatory response. The IKKβ/NF-κB inflammatory pathway might bridge gut microbiota, autophagy and insulin resistance. At the same time, it was observed that PU inhibited the IKKβ/NF-κB inflammatory pathway, regulated intestinal flora and upregulated liver autophagy. It was reported that in HFD-induced rats, blueberry supplementation regulated gut microbiota disturbance associated with improvements in systemic inflammation and insulin signaling (51). Resveratrol is a polyphenol which has been shown to be benefit to metabolic syndrome-related alterations in experimental animals, and the underlying mechanism might be its interaction with gut microbiota (52). A bioactive triterpenoid, celastrol, protected against high glucose-induced podocyte injury, inflammation and insulin resistance by restoring the autophagy pathway (53). An autophagy activator, rapamycin, decreased the inflammatory response and improved insulin resistance in type 2 diabetes rats through activation of autophagy (54). Therefore, we speculate that the effect of PU that inhibited inflammatory responses and improved insulin resistance was connected with regulating gut microbiota homeostasis and autophagy.

## Conclusion

In this study, it was concluded that PU can improve HFD-induced insulin resistance, hepatic glycolipid metabolism disorders and liver injury. The mechanism is possibly to inhibit the IKKβ/NF-κB inflammation pathway by regulating the gut microbiota and upregulating liver autophagy. We provided new evidence to understand the mechanism for development of insulin resistance, which was of important clinical significance for development of therapeutic approaches. PU had great potential and acted as an effective functional component for improving insulin resistance. The limitations are, first, we used an HFD-induced rodent model to mimic the development of human insulin resistance; however, the results cannot be applied to humans because human and rodent gut microbiota, and their physiology are different. In addition, although we found that PU improved the HFD-induced insulin resistance and that it was associated with improving the gut microbiota composition and gut microbiota disturbance-induced inflammatory response, a germ-free model is still needed to testify that the alteration of gut microbiota is the cause of inflammation and insulin resistance.

## Conflict of interest and funding

The authors had declared that no conflicts of interest exist. This research work was funded by Graduate Research and Innovation Project of Central South University (Grant No. 2018zzts854); Project of the Hunan Provincial Health Commission, China (Grant No. 20200123); and Natural Science Foundation of Hunan Province (Grant 2020JJ4778).
